# The effect of spatial resolution on deep learning classification of lung cancer histopathology

**DOI:** 10.1259/bjro.20230008

**Published:** 2023-08-15

**Authors:** Mitchell Wiebe, Christina Haston, Michael Lamey, Apurva Narayan, Rasika Rajapakshe

**Affiliations:** 1 University of British Columbia, Okanagan Campus, Kelowna, BC, Canada; 2 BC Cancer - Kelowna, Kelowna, BC, Canada; 3 University of Western Ontario, London, Ontario, Canada

## Abstract

**Objective:**

The microscopic analysis of biopsied lung nodules represents the gold-standard for definitive diagnosis of lung cancer. Deep learning has achieved pathologist-level classification of non-small cell lung cancer histopathology images at high resolutions (0.5–2 µm/px), and recent studies have revealed tomography–histology relationships at lower spatial resolutions. Thus, we tested whether patterns for histological classification of lung cancer could be detected at spatial resolutions such as those offered by ultra-high-resolution CT.

**Methods:**

We investigated the performance of a deep convolutional neural network (inception-v3) to classify lung histopathology images at lower spatial resolutions than that of typical pathology. Models were trained on 2167 histopathology slides from The Cancer Genome Atlas to differentiate between lung cancer tissues (adenocarcinoma (LUAD) and squamous-cell carcinoma (LUSC)), and normal dense tissue. Slides were accessed at 2.5 × magnification (4 µm/px) and reduced resolutions of 8, 16, 32, 64, and 128 µm/px were simulated by applying digital low-pass filters.

**Results:**

The classifier achieved area under the curve ≥0.95 for all classes at spatial resolutions of 4–16 µm/px, and area under the curve ≥0.95 for differentiating normal tissue from the two cancer types at 128 µm/px.

**Conclusions:**

Features for tissue classification by deep learning exist at spatial resolutions below what is typically viewed by pathologists.

**Advances in knowledge:**

We demonstrated that a deep convolutional network could differentiate normal and cancerous lung tissue at spatial resolutions as low as 128 µm/px and LUAD, LUSC, and normal tissue as low as 16 µm/px. Our data, and results of tomography–histology studies, indicate that these patterns should also be detectable within tomographic data at these resolutions.

## Introduction

Lung cancer is the leading cause of cancer death worldwide, with 1.8 million deaths and 2 million new cases in 2020.^1^ The most prevalent form of lung cancer is non-small cell which accounts for about 85% of all lung tumors.^2^ Adenocarcinoma (LUAD) and squamous-cell carcinoma (LUSC) represent the two main branches of non-small cell lung cancer (NSCLC) and have been shown to differ in their response to treatment, morphology, gene expression, and molecular composition.^3^ As such, the classification of lung cancer subtype is an important diagnostic process to inform the course of treatment.

A leading problem with lung cancer is that symptoms often present at advanced stages where treatment options are limited, or the disease is incurable.^4^ As such, effective screening procedures are crucial to improve early detection of lung cancer and improve patient outcomes. Low-dose CT (LDCT) is used to screen high-risk patients for possibly cancerous lesions, but it carries a high false-positive rate (25%).^5^ Lesions ≤ 3 mm, termed pulmonary nodules, can be detected with LDCT but their characterization poses a challenge for radiologists as assessment often requires follow-up imaging (such as conventional CT) to better visualize the lesion. Ultimately, to render a definitive diagnosis, invasive surgical procedures, such as biopsy, may be required.^6,7^ Such invasive follow-up exposes patients to the risk of further complications, and even death in extreme cases.^8^ Furthermore, as histological analysis may confirm the nodule as benign, there is a need to improve assessment of pulmonary nodules through non-invasive means.

The gold-standard for definitive diagnosis remains the analysis of biopsied tissue under light microscope by a trained pathologist; however, in recent years, the automated classification of biopsied tissue images with complex computational models has been investigated through deep learning.^9,10^ Deep learning involves allowing the computer model to experience a large data set and tune its internal parameters to produce a desired output. These models have been applied to classify lung cancer histopathology images from The Cancer Genome Atlas (TCGA) and have demonstrated pathologist-level classification performance [average area under the curve (AUC) = 0.97] at spatial resolutions of 0.5 and 2 µm/px.^11^ Human pathologists typically view histology slides within this range of resolution and may utilize even higher resolutions (up to around 0.25 µm/px) to provide a diagnosis. Deep learning models have also been shown capable of predicting LUAD *vs* LUSC from standard patient CT images at a spatial resolution of about 500 µm/px (AUC = 0.71^12^, AUC ≥0.86^13^).

Furthermore, pre-clinical tomographic imaging with micro-CT has correlated with histopathology of LUAD in mice (19 µm/px) and human dental caries (18 µm/px) among other domains.^14,15^ This suggests tomography and histology may achieve similar classification performance dependent on spatial resolution. Thus, towards understanding the spatial resolution required for non-invasive (CT) diagnosis, we investigated the effect of reducing image spatial resolution on the capacity of deep learning classification of gold-standard lung histopathology images.

## Methods and materials

### Data set and Pre-processing

We utilized the open-source TCGA lung cancer histology data set.^16,17^ The dataset comprised 2167 slides from 1010 patients (LUAD = 823, LUSC = 753, normal dense tissue = 591). Histology slides were first sorted into training (70%), validation (15%), and testing (15%) data sets among the three classes (LUAD/LUSC/normal). Slides were then partitioned into non-overlapping tiles (512 × 512 px) at 2.5 × magnification (4 µm/px) (openslide-python 1.1.1). Tiles with ≥50% white space (average RGB ≥ 220) were removed, yielding 18,949 image tiles. At this stage, each tile inherited the whole-slide label. As the tissue patterns occur with no inherent orientation, we applied 0^◦^ *,* 90^◦^ *,* 180^◦^ *,* and 270^◦^ rotations and mirroring to increase the training set size by a factor of eight (*n_train_* = 13*,*247→105*,*975). To ensure that models were not tested on data that may be directly related to the training data, all tiles from a given slide were allocated to the same set (either train, valid, or test), as were all slides from a given patient.

**Figure 1.**
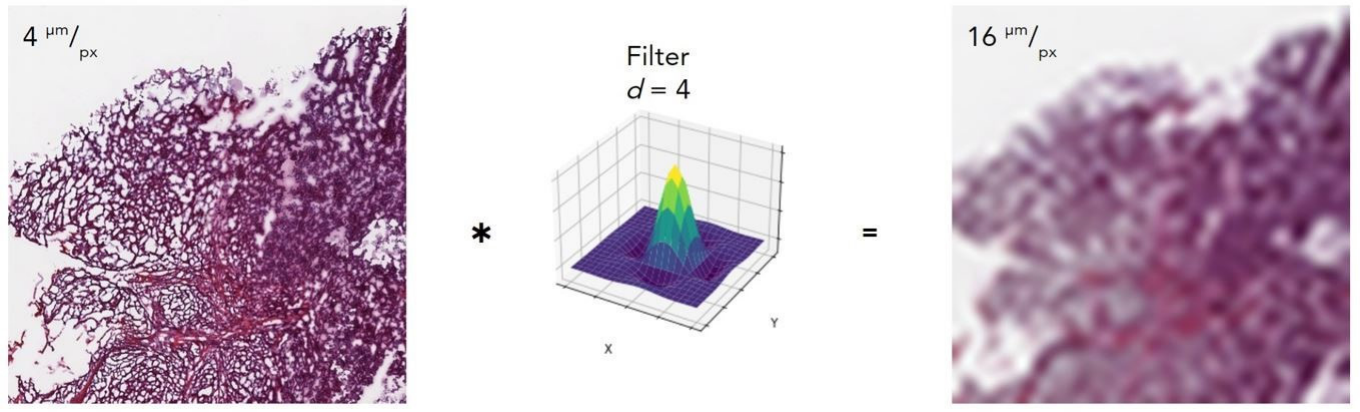
Low-pass filters reduce spatial resolution. The original 4 µm/px tile (left) is convolved with the *d* = 4 low-pass filter (middle), rendering a 16 µm/px tile (right). *d* = decimation factor.

To simulate data sets of reduced spatial resolution, Lanczos_3_ low-pass filters were applied to each tile within the original 4 µm/px data set (Figure 1). For this, two-dimensional filters were sampled from Equations 1 and 2 at values of x=(^n^/_d_) and y=(^m^/_d_), where *n* and *m* are sample numbers (integers), and *d* is the decimation factor.^18^

(1) Lanczos3x=sincπxsincπx3,ifx§amp;lt;30,otherwise

(2) $${Lanczos}_{3}(x,y)={Lanczos}_{3}(x)\times {Lanczos}_{3}(y)$$

We followed an *n^2^* rule to create filters with decimation factors of *d* = 2, 4, 8, 16, and 32 (Supplementary Figure ). When applied to the original 4 µm/px data set, this produced separate data sets with spatial resolutions of 8, 16, 32, 64, and 128 µm/px, respectively.^18,19^ These spatial resolutions were chosen as they are below what has been investigated in previous research and are within the range of what is achievable with micro-CT technology.^20^ With this method, the size of each data set and image dimensions remained unaffected. The only variation between data sets was the level of spatial resolution decimation.

Supplementary Figure 1.

Supplementary Figure 2.

Supplementary Figure 3.

### Deep learning pipeline

**Figure 2.**
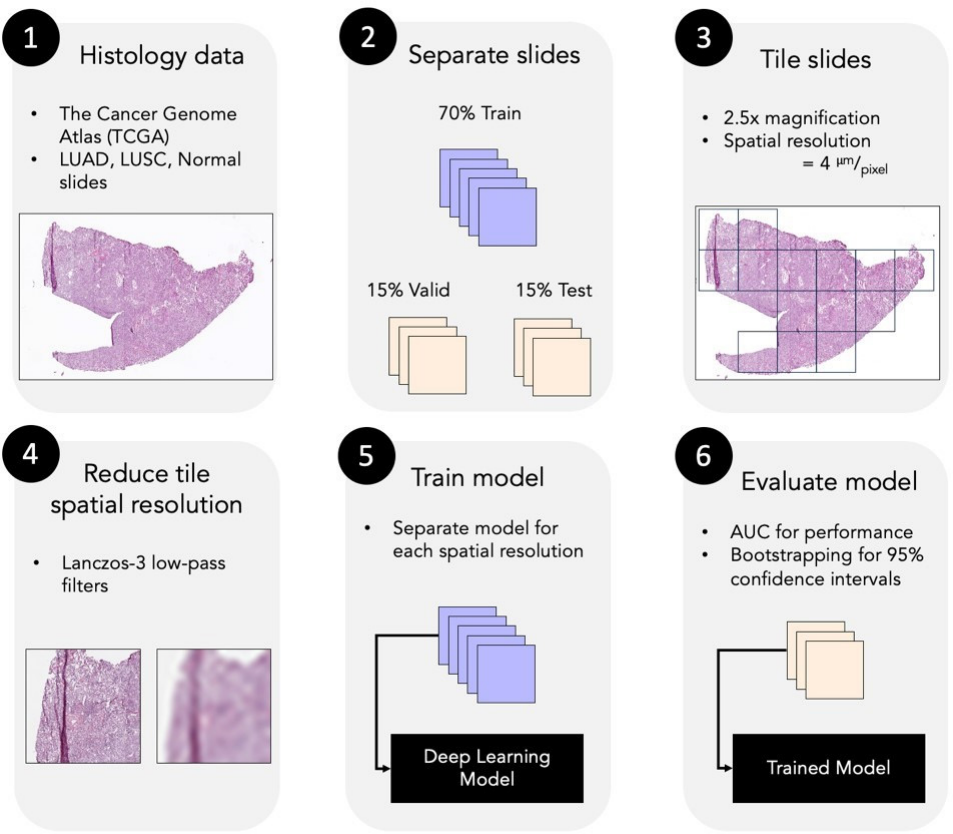
Deep learning pipeline. (1) Histology slides from TCGA were acquired. (2) Slides were separated into train, validation, and test sets. (3) Slides were partitioned into smaller tiles at 2.5× magnification (4 µm/px). (4) Separate data sets of reduced resolution were generated by applying low-pass filters. (5) Separate models were trained on each data set. (6) Model performance was evaluated by ROC curves and AUC on a per-slide basis. AUC, area under the receiver operating characteristics curve; LUAD, adenocarcinoma; LUSC, squamous cell carcinoma; AUC, area under the receiver operating characteristics curve; ROC, receiver operator characteristic; TCGA, The Cancer Genome Atlas.

The deep learning pipeline is illustrated in Figure 2. Inception-v3 was fully trained from scratch, allowing all convolutional layers to be updated, with hyperparameters as used by Coudray et al.^11^ (Optimizer = RMSProp, learning rate = 0.1, weight decay = 0.9, momentum = 0.9, ε = 1.0). Separate inception-v3 instances were trained for each spatial resolution data set (Ex: model instance trained and tested on 4 µm/px only). Models were trained for 400,000 steps on the task of differentiating LUAD, LUSC, and normal tissue with a batch size of 32 (1 step = 1 batch processed). Training each model on NVIDIA GeForce RTX 2080 Ti GPU (11 GB VRAM) took approximately 48 h (python 3.6.8, tensorflow-gpu 1.9.0).

Models were check-pointed every 25,000 steps, and the best performing checkpoint on the validation set was evaluated on the test set. Predictions were obtained for each tile within the test set and the predicted scores for each tile from a given slide were averaged to obtain whole-slide predictions. Slide-level performance was evaluated by receiver operating characteristic curves (ROCs) and area under the curve (AUC) (scikit-learn 0.24.2, scipy 1.5.4). Bootstrapping was performed 2000 times to determine 95% confidence intervals in AUC metrics.

## Results

### LUAD *vs* LUSC *vs* normal tissue

**Figure 3.**
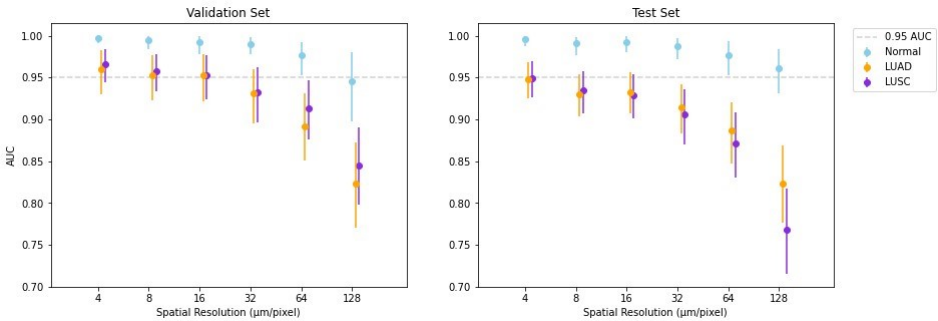
Model performance to predict LUAD/LUSC/normal dense tissue. AUC of validation and test sets at spatial resolutions of 4, 8, 16, 32, 64, and 128 µm/px. Error bars represent 95% confidence intervals as determined by bootstrapping. Normal (blue), LUAD (orange), LUSC (purple). AUC, area under the receiver operating characteristics curve; LUAD, adenocarcinoma; LUSC, squamous-cell carcinoma.

Figure 3 shows the model ability to predict each of LUAD, LUSC, and normal tissue at each resolution tested. AUCs are plotted for each tissue type and were compared against an arbitrary performance threshold of AUC = 0.95. Full ROC curves are provided as Supplementary Figures. The model demonstrated overall greater ability to discriminate normal lung from either cancer type, while these two cancer types were more difficult to differentiate from one another. In general, as spatial resolution decreased, model classification performance decreased. Specifically, at the 95% confidence intervals, model predictions for all three classes overlapped with the AUC = 0.95 threshold at resolutions of 4, 8, and 16 µm/px. Below these resolutions, model classification of LUAD and LUSC decreased, indicating that important discriminating patterns were being obscured. Notably, normal tissue could be differentiated from the two cancer types as low as 128 µm/px.

### Tiled heatmaps

**Figure 4.**
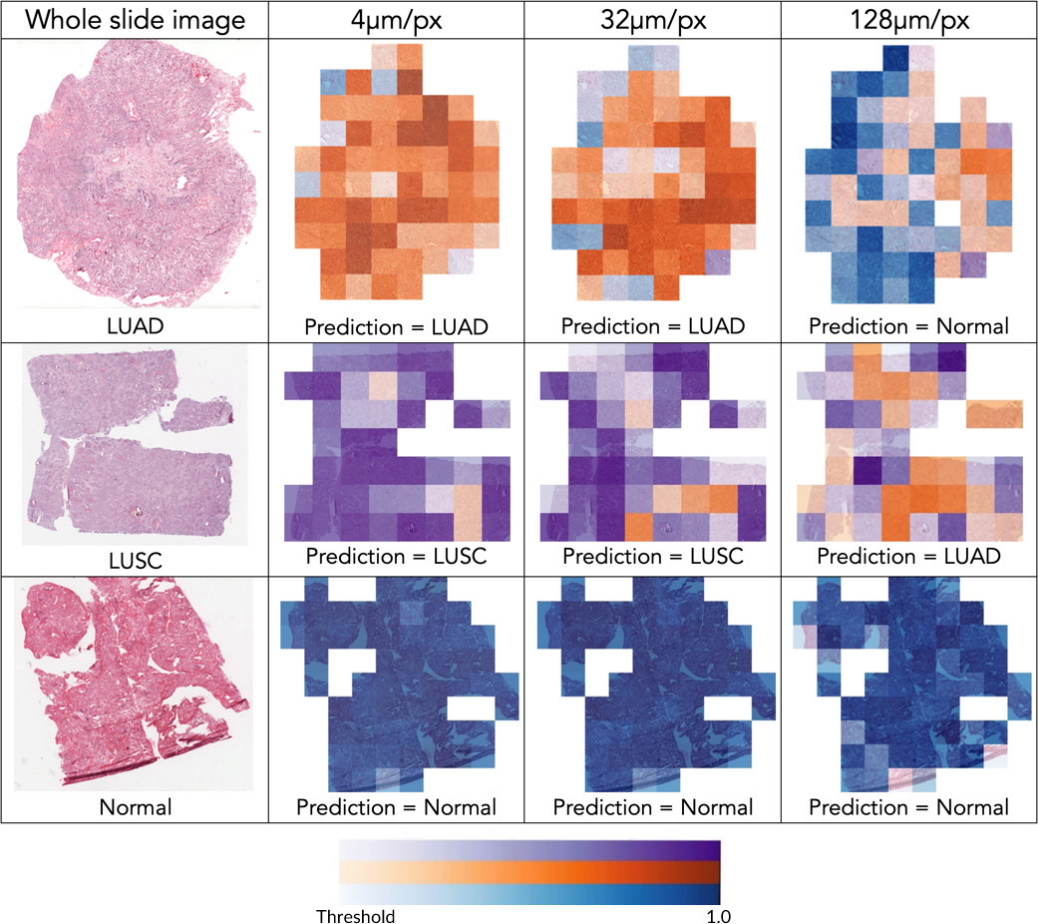
Tiled heatmaps and model predictions at various spatial resolutions. Normal (blue), LUAD (orange), and LUSC (purple) tile predictions are labeled. Color-bar provides reference for the magnitude of prediction above the decision threshold. Normal prediction = blue, LUAD prediction = orange, and LUSC prediction = purple. LUAD, adenocarcinoma; LUSC, squamous-cell carcinoma

We generated tiled heatmaps of whole slides to gain a qualitative understanding of how spatial resolution was affecting the model’s performance. Each tile was assigned a color according to the model’s prediction, with darker shades representing predictions of greater magnitude than lighter ones. Figure 4 shows that as spatial resolution decreased, an increased number of LUAD and LUSC tiles were misclassified, affecting the final classification of the whole slide. Normal tile classification was less affected by spatial resolution as shown in Figure 4. In general, lower spatial resolution heatmaps were more likely to deviate from higher resolution heatmaps by making incorrect tile predictions of greater magnitude.

## Discussion

Our work is distinct in that we purposefully degraded image resolution to understand limits of deep learning for histopathology classification and recommend spatial resolutions that could be useful for a downstream task (*i.e.* non-invasive classification via high-resolution CT). We showed that deep learning can differentiate between LUAD, LUSC, and normal TCGA histology slides at spatial resolutions of 4–16 µm/px. All three tissues can be classified to the performance threshold of AUC ≥0.95 at spatial resolutions achievable with micro-CT imaging (typical range 5–100 µm/px^20^). Our data and the results of previous histology-micro-CT correlation studies^14,15^ suggest that similar classification performance should be possible within tomographic data at these spatial resolutions. Furthermore, it is possible that current CT scanners could be adapted to image at these resolutions, an advancement that would repurpose current equipment to benefit both rural and urban cancer centers in terms of non-invasive lesion classification.

We selected the inception-v3 model based on its prior success at classifying different resolutions of lung histopathology images^11^ and aimed to document its performance if lower-spatial resolutions were the only inputs available. This represents a limitation of our research as other deep learning algorithms may perform differently. A comparison across different deep learning models is beyond the scope of this work, but remains a potential area of investigation for future research.

At 4 and 8 µm/px, our results are comparable to Coudray et al,^11^ who investigated this problem at 0.5 and 2 µm/px. Notably, a decrease in spatial resolution from 0.5 to 8 µm/px did not affect classification performance within the TCGA data set. However, at 16 µm/px (the lowest spatial resolution that all tissues could be classified within tolerance of the threshold) our model predictions are slightly worse than Coudray et al for LUSC, indicating that important LUSC patterns were becoming obscured at this resolution.

Our method to understand the effect of reduced spatial resolution on lung cancer histology classification is consistent with Sabottke et al^21^ who also investigated the effect of image resolution on classification of radiography images. Sabottke et al reduced image resolution by altering image dimensions with bilinear interpolation, whereas we present the use of low-pass filters to reduce spatial resolution without manipulating image dimensions. Thus, the low-pass filter method extends approaches available for studies requiring a reduction in image resolution.

Our results are inherently limited by the TCGA data set as there is no guarantee that it was completely representative of all histological manifestations of lung tissues and may not fully represent the range of features encountered by pathologists. As a result, our method may not uphold the same performance on an independent data set. Future work should investigate the generalizability of our methodology to classify lung histopathology images from external data sets. Another limitation of the TCGA data set is that labels are only provided at the slide-level. If labels could be applied on a tile-level, it is possible that performance could be improved. Moreover, it is possible that model performance at all spatial resolutions could be improved if a more extensive data set were available.

Our results indicate that increased spatial resolution improved tissue classification within histology slide data. Building on findings from Chaunzwa et al,^12^ who have demonstrated the potential for classifying lung cancer histopathology from two-dimensional sections of CT images (AUC = 0.71 for LUAD *vs* LUSC), our work suggests that improved classification performance should be possible with higher resolution tomography data. This could aid in describing morphological disease characteristics and patient risk stratification. Moreover, Wang et al^13^ demonstrated the utility of using three-dimensional CT data for classifying lung cancer histology, as AUCs ≥ 0.86 for predicting benign tumours, LUAD, and LUSC among other tissue types were achieved. Despite the lower spatial resolution of CT images (500 µm/px), their model performed comparably to our 64 µm/px classifier for LUAD and LUSC, suggesting that capturing tumor spatial relations in all three dimensions contributes to classification. As our histology data are inherently two-dimensional and CT data is three-dimensional, our model predictions might be improved with the use of tomographic data at the spatial resolutions investigated.

## Conclusion

In this work, the effect of spatial resolution on classification of gold-standard lung cancer histology slides was investigated with a deep convolutional neural network (inception-v3). We demonstrated that classification performance decreased as spatial resolution decreased, but that normal dense lung tissue could be differentiated from cancerous lung tissue at spatial resolutions as low as 128 µm/px. LUAD and LUSC tissues could be differentiated at higher spatial resolutions ranging from 4 to 16 µm/px.
