# Supplementary figures and images for: The effect of spatial resolution on deep learning classification of lung cancer histopathology
Source: BJR Open. 2023 Aug 15;5(1):20230008. doi: 10.1259/bjro.20230008 (PMC10636338; doi:10.1259/bjro.20230008)

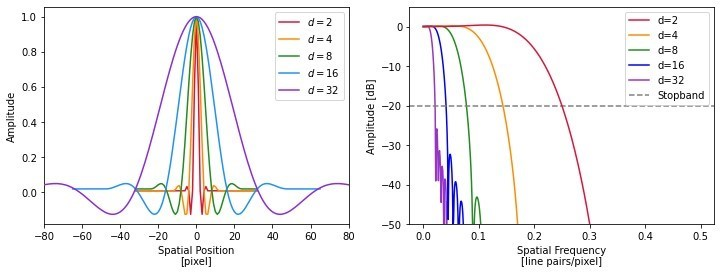

Supplement: Supplementary file 1 — Supplementary Figure 1. [file bjro.20230008.suppl-01.tiff]

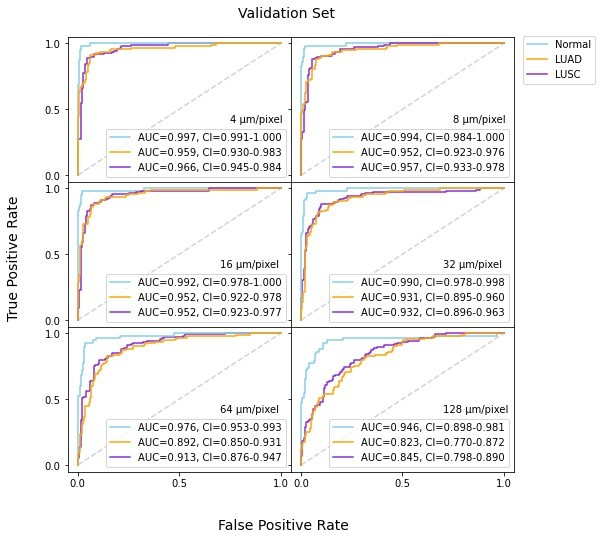

Supplement: Supplementary file 2 — Supplementary Figure 2. [file bjro.20230008.suppl-02.tiff]

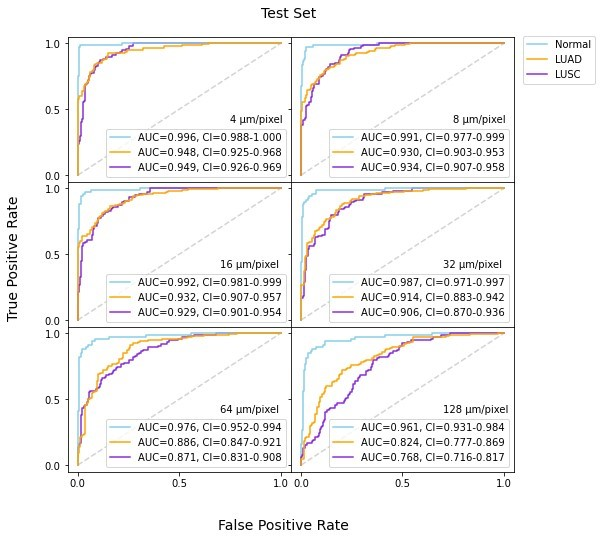

Supplement: Supplementary file 3 — Supplementary Figure 3. [file bjro.20230008.suppl-03.tiff]
